# TBX5-AS1, an enhancer RNA, is a potential novel prognostic biomarker for lung adenocarcinoma

**DOI:** 10.1186/s12885-021-08517-w

**Published:** 2021-07-09

**Authors:** Lin Cheng, Tong Han, Bolin Chen, Kechao Nie, Weijun Peng

**Affiliations:** 1 Department of Integrated Traditional Chinese & Western Medicine, The Second Xiangya Hospital, Central South University, No.139 Middle Renmin Road, Changsha, Hunan 410011 People’s Republic of China; 2 Department of General Surgery, The Second Xiangya Hospital, Central South University, Changsha, 410011 Hunan China; 3 Department of Thoracic Medical Oncology, Hunan Cancer Hospital/the affiliated Cancer Hospital of Xiangya school of Medicine, Central South University, Changsha, 410013 Hunan China; 4 Department of Endocrinology, The First Affiliated Hospital of Guangzhou University of Chinese Medicine, Guangzhou, 510405 Guangdong China

**Keywords:** TBX5-AS1, Enhancer RNA, LUAD, TBX5, Prognostic biomarker

## Abstract

**Background:**

Enhancer RNAs (eRNAs) are demonstrated to be closely associated with tumourigenesis and cancer progression. However, the role of eRNAs in lung adenocarcinoma (LUAD) remains largely unclear. Thus, a comprehensive analysis was constructed to identify the key eRNAs, and to explore the clinical utility of the identified eRNAs in LUAD.

**Methods:**

First, LUAD expression profile data from the Cancer Genome Atlas (TCGA) dataset and eRNA-relevant information were integrated for Kaplan-Meier survival analysis and Spearman’s correlation analysis to filtered the key candidate eRNAs that was associated with survival rate and their target genes in LUAD. Then, the key eRNA was selected for subsequent clinical correlation analysis. KEGG pathway enrichment analyses were undertaken to explore the potential signaling pathways of the key eRNA. Data from the human protein atlas (HPA) database were used to validate the outcomes and the quantitative real time-polymerase chain reaction (qRT-PCR) analysis was conducted to measure eRNA expression levels in tumor tissues and paired normal adjacent tissues from LUAD patients. Finally, the eRNAs were validated in pan-cancer.

**Results:**

As a result, TBX5-AS1 was identified as the key eRNA, which has T-box transcription factor 5 (TBX5) as its regulatory target. KEGG analysis indicated that TBX5-AS1 may exert a vital role via the PI3K/AKT pathway, Ras signaling pathway, etc. Additionally, the qRT-PCR results and the HPA database indicated that TBX5-AS1 and TBX5 were significantly downregulated in tumour samples compared to matched-adjacent pairs. The pan-cancer validation results showed that TBX5-AS1 was associated with survival in four tumors, namely, adrenocortical carcinoma (ACC), LUAD, lung squamous cell carcinoma (LUSC), and uterine corpus endometrial carcinoma (UCEC). Correlations were found between TBX5-AS1 and its target gene, TBX5, in 26 tumor types.

**Conclusion:**

Collectively, our results indicated that TBX5-AS1 may be a potential prognostic biomarker for LUAD patients and promote the targeted therapy of LUAD.

**Supplementary Information:**

The online version contains supplementary material available at 10.1186/s12885-021-08517-w.

## Background

Lung cancer is recognized as one of the most common malignant carcinomas, and ranks first for morbidity and mortality among all malignant tumors in the world. Non-small cell lung cancer (NSCLC) is the most frequently diagnosed type of lung cancer, and comprises two major types—adenocarcinoma and squamous carcinoma [1, 2]. Because the early symptoms of lung cancer aren’t clear and are confused easily with those of other respiratory diseases, the rates of misdiagnosis and missed diagnosis are high, and the majority of patients are diagnosed at an intermediate or late stage, at which the effective treatment options are limited and the prognosis is usually poor. In recent years, with the development of gene detection technology and targeted therapies, great strides have been made in the treatment and prognosis of LUAD. However, the 5-year overall survival is still less than 20% [3, 4], highlighting the urgent need to identify effective prognosis-related biomarkers for LUAD; evaluating the prognosis of LUAD patients through biomarkers in the early stage of the disease is of great importance to improving the prognosis and survival rate of these patients.

Over the past decade, the rapid application and development of high-throughput sequencing technology has opened a new window for noncoding RNAs, and accumulating evidence has shown that the occurrence and development of a series of major diseases are related to noncoding RNA dysregulation [5–7]. Among these, RNAs generated from enhancers (enhancer RNAs; eRNAs) have gained substantial interest owing to their underlying roles in the mediation of gene transcription and enhancer functions, and their frequent overlap with noncoding risk loci associated with diseases [8–11]. Enhancers are key *cis*-regulatory elements that promote the expression of eukaryotic genes [12, 13]. eRNAs are 500–5000 bp-long RNAs produced through enhancer transcription [14]. They can activate enhancer activity independently, and can also combine with other protein factors to promote the formation of the enhancer-promoter loop, thereby activating the expression of downstream genes [15–17]. eRNA dysregulation can affect biological processes such as the cell cycle and the growth of cancer cells, or change the expression of target genes, suggesting that eRNAs may serve as novel targets for tumour treatment [15, 18, 19]. A recent study found that, mutations in the eRNA element of *ACTRT1* in basal cell carcinoma can weaken the enhancer’s activity as well as the expression of *ACTRT1*, resulting in the aberrant activation of Hedgehog signaling and, consequently, the promotion of carcinogenesis and tumour development [20]. In T-cell acute lymphoblastic leukemia (T-ALL), it was also reported that the eRNA ARIEL could recruit mediator proteins to the *ARID5B* enhancer, thereby boosting enhancer-promoter interactions and activating the expression of *ARID5B*. These activities led to the aberrant activation of the *TAL1*-induced transcriptional program and myc oncogenic signals [21]. *NET1* is an oncogene of key importance in breast cancer. Related research has demonstrated that the *NET1-*associated eRNA (NET1e) could upregulate the expression of its target gene and promote breast cancer progression. In addition, overexpression of NET1e in situ can lead to the resistance of breast cancer cells to BEZ235 and octreotide [22]. Additionally, a separate research discovered an eRNA, AP001056.1, is able to serve to be a prognostic marker for head and neck squamous cell carcinoma (HNSCC) [23]. However, to date, no study has investigated the potential of using eRNAs as biomarkers to predict the survival of patients with LUAD.

Herein, we aimed at identifying prognostic eRNAs as well as their target genes within LUAD for predicting LUAD patients’ prognosis. Our results may facilitate the understanding of the potential value of the clinical application of LUAD-associated eRNAs in prognosis stratification and promote the targeted therapy of LUAD.

## Materials and methods

### Identification of predictive eRNAs in LUAD by comprehensive data analysis

Clinical data, RNA expression profile data, and survival information for 33 cancer types were downloaded from the TCGA database. All RNA expression profile data were transformed to log2 (FPKM+ 1). Ensemble transcript IDs were con-verted to their corresponding GENCODE using GTF annotation files for humans. We searched and comprehensively analysed the eRNA source literature to obtain the relevant eRNA information. The human GTF files were used to transform eRNA transcript IDs into gene symbols and the expression profiles of the eRNAs were extracted from the RNA expression profile of lung adenocarcinoma. Then, we combined the eRNA expression matrix with the lung adenocarcinoma survival data using the limma R software package.

The survival-associated eRNAs were screened using Kaplan-Meier methodology, taking the false discovery rate (FDR) adjusted *P* < 0.05 as standard cut-off values, we selected eRNAs that met this condition as survival-associated eRNAs. The patients were classified into low- and high-expression groups according to the median expression of each eRNA. Differences in survival were compared between the two groups using *p* < 0.05 as the cut-off and a survival curve for the eRNAs that met the conditions was drawn. Then, Spearman’s correlation analysis was performed to obtain candidate key eRNAs related to survival and associated with target genes with roles in LUAD; statistical significance was considered at cor > 0.4 and *p* < 0.001.

### Gene enrichment analysis

Besides the predicted targets, Pearson’s correlation analysis was performed to identify other genes in LUAD that were significantly associated with the key eRNA (cor > 0.4, and *p* < 0.001). The target genes of the key eRNA were converted into their corresponding gene IDs for further analysis using the org. Hs.eg.db R software package. To investigate which functions and pathways were affected by the eRNA in tumorigenesis and metastasis, the clusterProfiler package in R was used for the pathway enrichment analyses of Kyoto Encyclopedia of Genes and Genomes (KEGG) and Gene Ontology (GO) www.kegg.jp/kegg/kegg1.html) [24–26] of the key eRNA target genes. Enriched GO terms with an adjusted *p*-value < 0.001 were considered as relevant biological processes.

### Clinical LUAD sample collection

A total of 10 LUAD tumor tissues and para-tumor samples were collected from patients diagnosed with LUAD according to pathological analysis and who were undergoing surgery at the Second Xiangya Hospital of Central South University. After tumor resection, the tumor and adjacent tissues were immediately frozen in liquid nitrogen and stored at − 80°Cuntil total RNA extraction. All patients gave their informed consent for inclusion before surgery. The study was conducted in accordance with the Declaration of Helsinki, and the protocol was approved by the Ethics Committee of the Second Xiangya Hospital of Central South University (protocol code 2020038 and 07/01/2020).

### Quantitative real-time polymerase chain reaction (qRT-PCR) validation

TRIzol reagent was used to extract total RNA from tissue samples (Invitrogen, GrandIsland, NY, USA). The quality and quantity of RNA were estimated by NanoDropND-1000 (Thermo Scientific, Waltham, MA, USA), while the integrity of RNA were assessed by standard denaturing agarose gel electrophoresis. Then, the RNA was reverse-transcribed into cDNA by adopting SuperScript III Reverse Transcriptase (Invitrogen) in accordance with the instructions of the manufacturer. A 2× PCR Master Mix (Arraystar) and an Applied Biosystems ViiA 7 Real-Time PCR System were adopted for qRT-PCR following the instructions of the manufacturer. The relative eRNA and mRNA expression levels were calculated using the 2 − ΔΔCt method. The primers used and the sequences are listed in Table 1 and Table 2. The data represent the means of three experiments.

**Table 1:** Primers designed for qRT-PCR validation of eRNA

Name	Bidirectional primer sequence	Tm (°C)	Product length (bp)
β-actin(H)	F:5′ GTGGCCGAGGACTTTGATTG3’	60	73
	R:5′ CCTGTAACAACGCATCTCATATT3’		
TBX5-AS1	F:5′ CGCAGTGGTGGATGCTCG 3’	60	189
	R:5′ CTCGGCTCAGAGGTCAAGTAGG 3’

**Table 2:** Primers designed for qRT-PCR validation of mRNA

Name	Bidirectional primer sequence	Tm (°C)	Product length (bp)
β-actin(H)	F:5′ GTGGCCGAGGACTTTGATTG3’	60	73
	R:5′ CCTGTAACAACGCATCTCATATT3’		
TBX5	F:5′ GGTCTCTTTTGGTGGTCCTTTT 3’	60	195
	R:5′ TCAGGCTCCAGAGGCGTGT 3’		

### Human protein atlas analysis

The human protein atlas (HPA; https://www.Proteinatlas.org/) comprises an atlas of human protein expression patterns in normal and tumor tissues. In this study, we assessed the expression of T-box transcription factor 5 (TBX5) in the protein expression module of the HPA database and analyzed the immunohistochemical staining results for TBX5 in both tumor and normal tissues (antibody: HPA008786).

### Verification in pan-Cancer

First, the expression data of TBX5-AS1 and its target gene TBX5 in pan-cancer were obtained using the R limma package, and then the expression matrix was combined with the pan-cancer survival data. The samples were classified into low- and high-expression groups according to the median value of the expression of TBX5-AS1, and then the Kaplan–Meier method was used to compare the survival difference between the two groups. *p* < 0.05 was considered statistically significant. A survival curve was plotted for TBX5-AS1 in tumours that met the criteria. Spearman’s coefficient was used to test the cor-relation between TBX5-AS1 and its corresponding target genes TBX5 in pan-cancer. A correlation coefficient > 0.4 and a *p*-value < 0.001 were considered statistically significant.

## Results

### Screening the key eRNA in LUAD

**Fig. 1:**
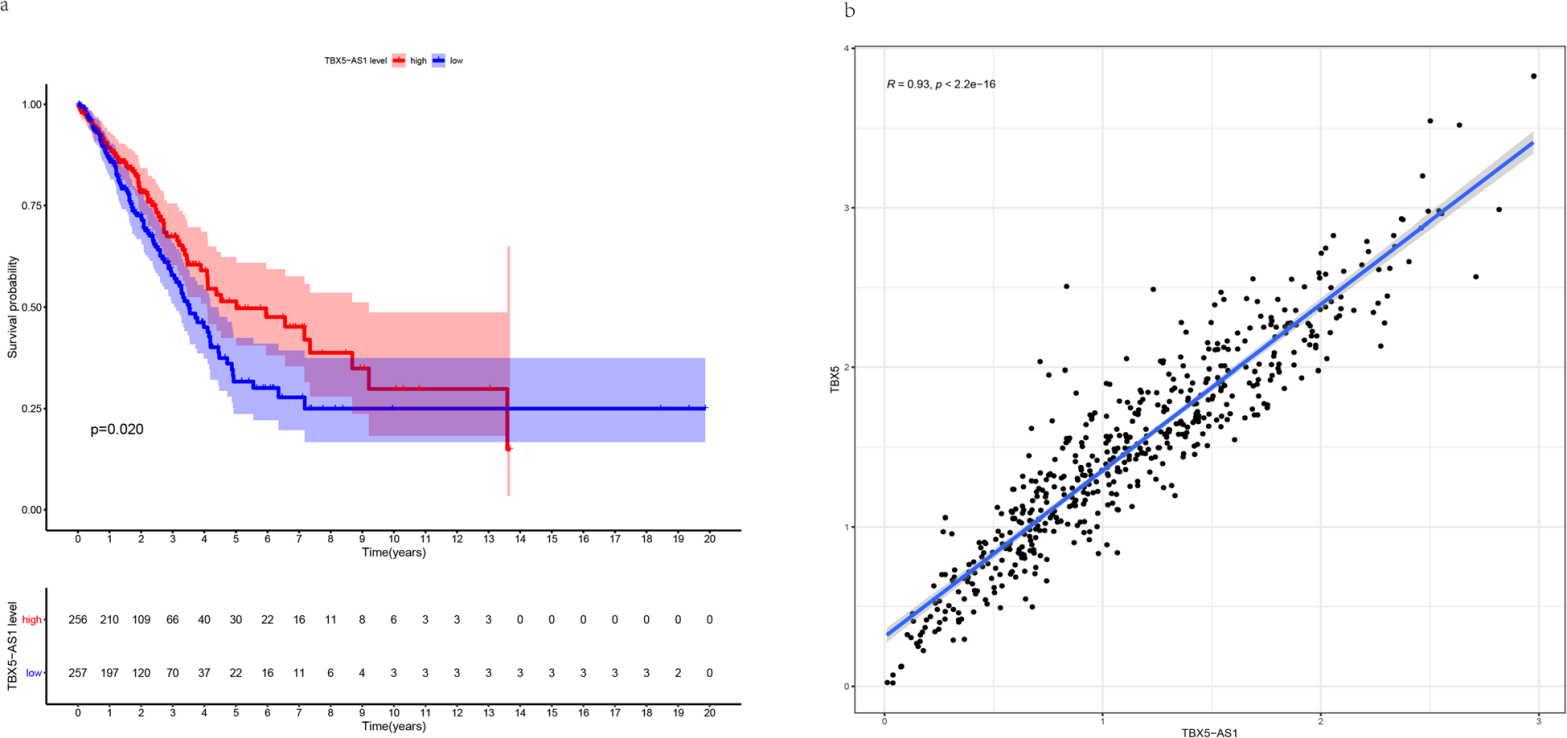
**a** Kaplan–Meier survival curves for TBX5-AS1. **b** A scatter plot showing the correlation between the TBX5 proportion and TBX5-AS1 expression (*p* < 0.05)

**Fig. 2:**
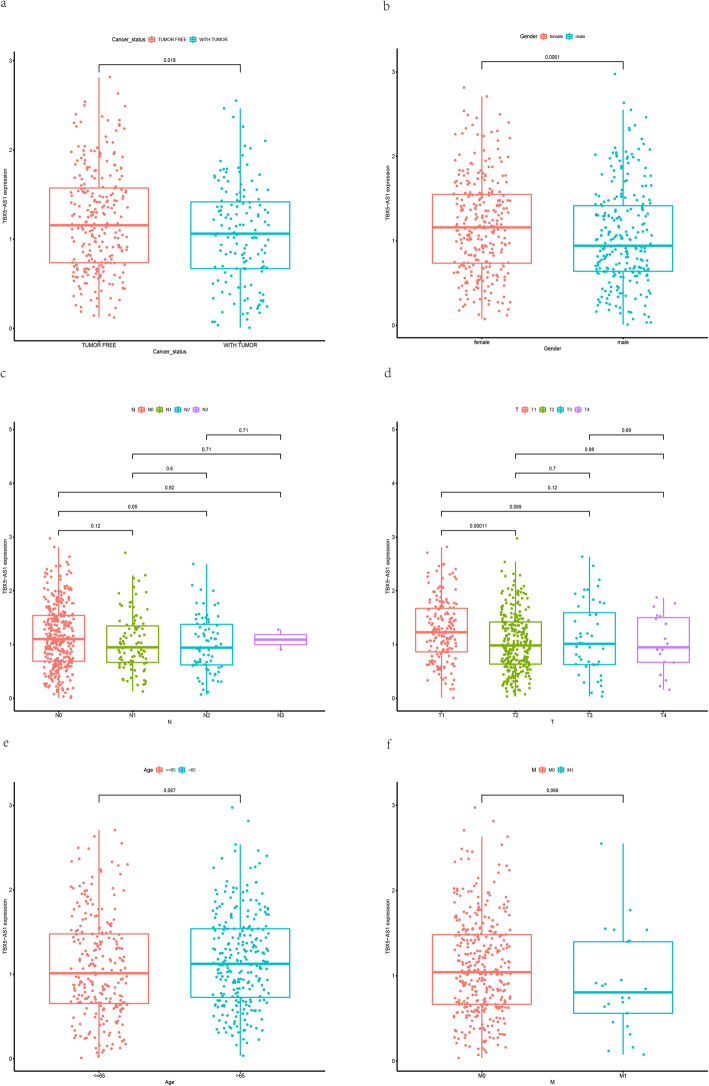
The relationship between TBX5-AS1 and clinical features; **a** cancer status (*p* = 0.018); **b** gender (*p* = 0.0061); **c** N stage (N0 vs. N2, *p* = 0.05); **d** T stage (T1 vs. T2, *p* = 0.00011); **e** age (*p* > 0.05); and (**f**) M stage (*p* > 0.05)

Using the Kaplan-Meier method, eRNAs with *P*-values adjusted according to the FDR cut-off of < 0.05 were extracted as survival-related eRNAs in LUAD. A total of 174 eRNAs were identified (Additional file 1: Table S1). Spearman’s correlation was then used to screen these 174 eRNAs to identify those with a significant correlation with their target genes that were associated with LUAD. Only 76 eRNAs met the condition (Spearman’s rank correlation coefficient *r* > 0.40, *p* < 0.001; Additional file 1: Table S2). Of these, TBX5-AS1 exhibited the highest cor value, and was therefore considered to be the most relevant eRNA associated with its target gene. Kaplan–Meier curve results for TBX5-AS1 showed that patients in the low-expression group survived longer than those in the high-expression group (*p* = 0.02998; Fig. 1a). In addition, there was a positive correlation between TBX5-AS1 and its target gene TBX5 (*r* = 0.89973, *p* = 2.2 × 10–15; Fig. 1b). We further investigated the connections between the clinical features of LUAD patients and TBX5-AS1 expression and found that the expression level of TBX5-AS1 in LUAD was significantly linked to T stage (T1 vs. T2, *p* = 0.00011), N stage (N0 vs. N2, *p* = 0.05), sex (*p* = 0.0061), and cancer status (*p* = 0.018) (Fig. 2a–d); however, other clinical characteristics, such as age and M stage, had no clear correlation with TBX5-AS1 expression (*p* > 0.05) (Fig. 2e, f).

### Gene enrichment analysis

**Fig. 3:**
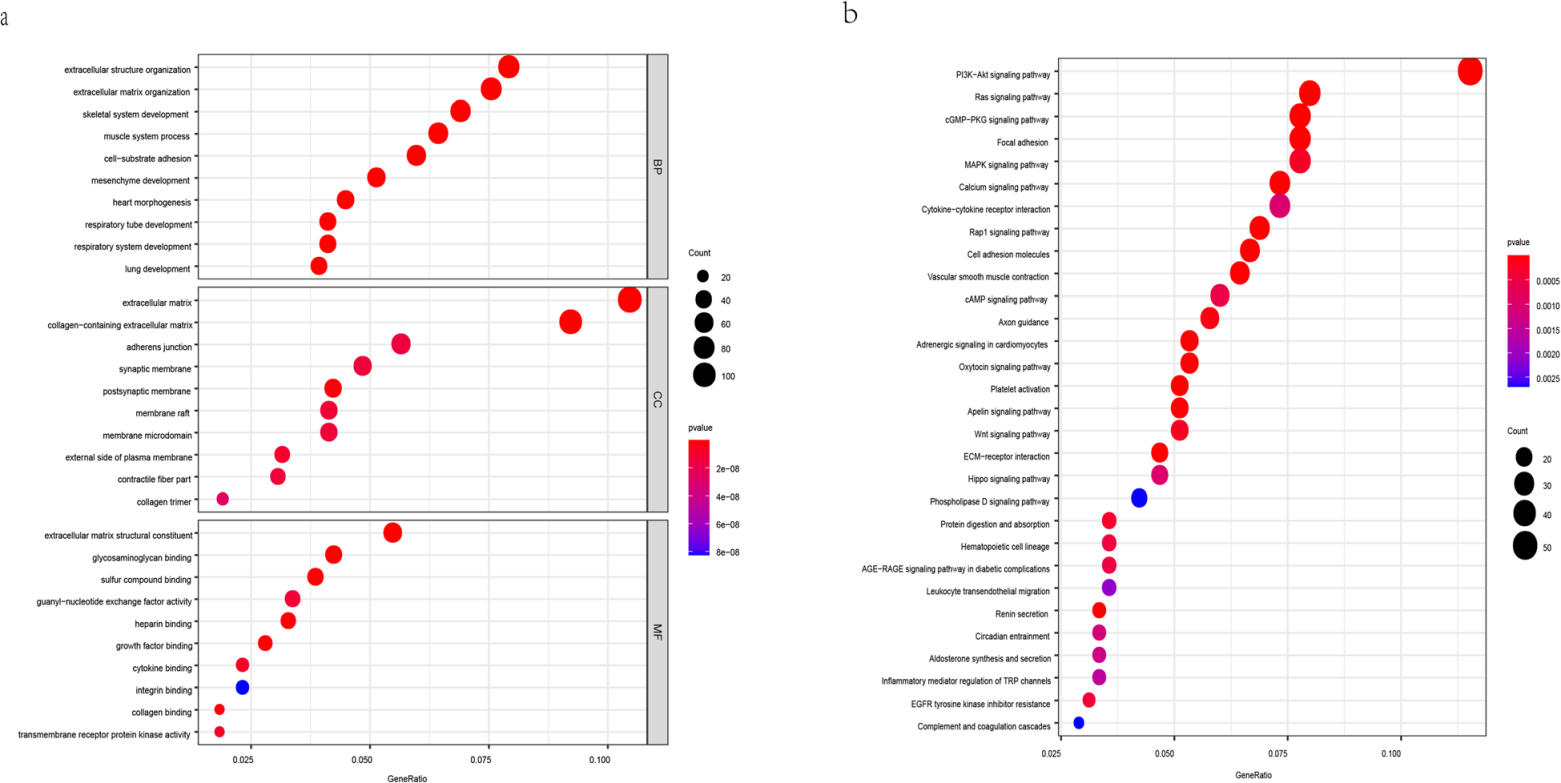
**a** Biological process (BP), cellular component (CC), and molecular function (MF) in Gene Ontology (GO) enrichment analysis. **b** The top 30 enriched KEGG pathways

A total of 1407 transcripts presented a significant correlation with TBX5-AS1 (*p* < 0.05), including TBX5. GO enrichment analysis and KEGG pathway analysis of the 1407 target genes provided the basis for the biological study of these genes. The top 10 terms for molecular function (MF), biological process (BP) and cellular component (CC) are summarized in Fig. 3a. In BP, the terms were mainly related to extracellular matrix organization and extracellular structure organization; in CC, they were related to extracellular matrix, collagen-containing extracellular matrix, postsynaptic membrane, and adherens junction, among others. In MF, term enrichment mainly involved extracellular matrix structural constituent and glycosaminoglycan binding. The intensity of the colors indicates the *p*-value (stronger colors indicate lower *p*-values)—the lower the p-value, the more significant the GO term. The top 30 pathways are summarized in Fig. 3b. KEGG enrichment analysis showed that the most significantly enriched biological process was “PI3K/AKT signaling pathway” (adjusted *p* < 0.001). The PI3K/AKT signaling pathway-related genes with a Spearman’s correlation coefficient > 0.6 are depicted in Table 3.

**Table 3:** List of the PI3K/AKT signaling pathway-related genes associated with TBX5-AS1 expression (*r* > 0.600, *p* < 0.001)

Gene	cor	Gene	cor	Gene	Cor
ITGA8	0.817	TNXB	0.574	FGF1	0.458
LAMA2	0.785	VWF	0.566	THBS1	0.443
PDGFRA	0.699	F2R	0.56	RBL2	0.442
HGF	0.696	COL6A3	0.533	JAK1	0.438
GHR	0.696	ITGA4	0.531	FGFR2	0.438
ANGPT1	0.693	NTF3	0.53	ITGA10	0.432
FGF10	0.692	VEGFD	0.527	PIK3R5	0.428
PDGFRB	0.675	IL7R	0.518	PTEN	0.42
COL6A5	0.646	ITGA1	0.517	ERBB4	0.419
GNG2	0.623	PIK3CG	0.508	NGFR	0.418
FGF7	0.622	TLR4	0.491	COL4A2	0.408
ITGA9	0.619	TNR	0.486	FGFR1	0.407
TEK	0.6	ANGPT4	0.482	PPP2R2B	0.407
COL6A6	0.598	MAGI2	0.478	FLT4	0.405
LAMA4	0.597	FLT3	0.474	COL4A4	0.401
TNN	0.589	AKT3	0.47	PIK3R6	0.401
PIK3R1	0.584	GNG7	0.463		
FGF2	0.575	CCND2	0.462		

### Data validation

**Fig. 4:**
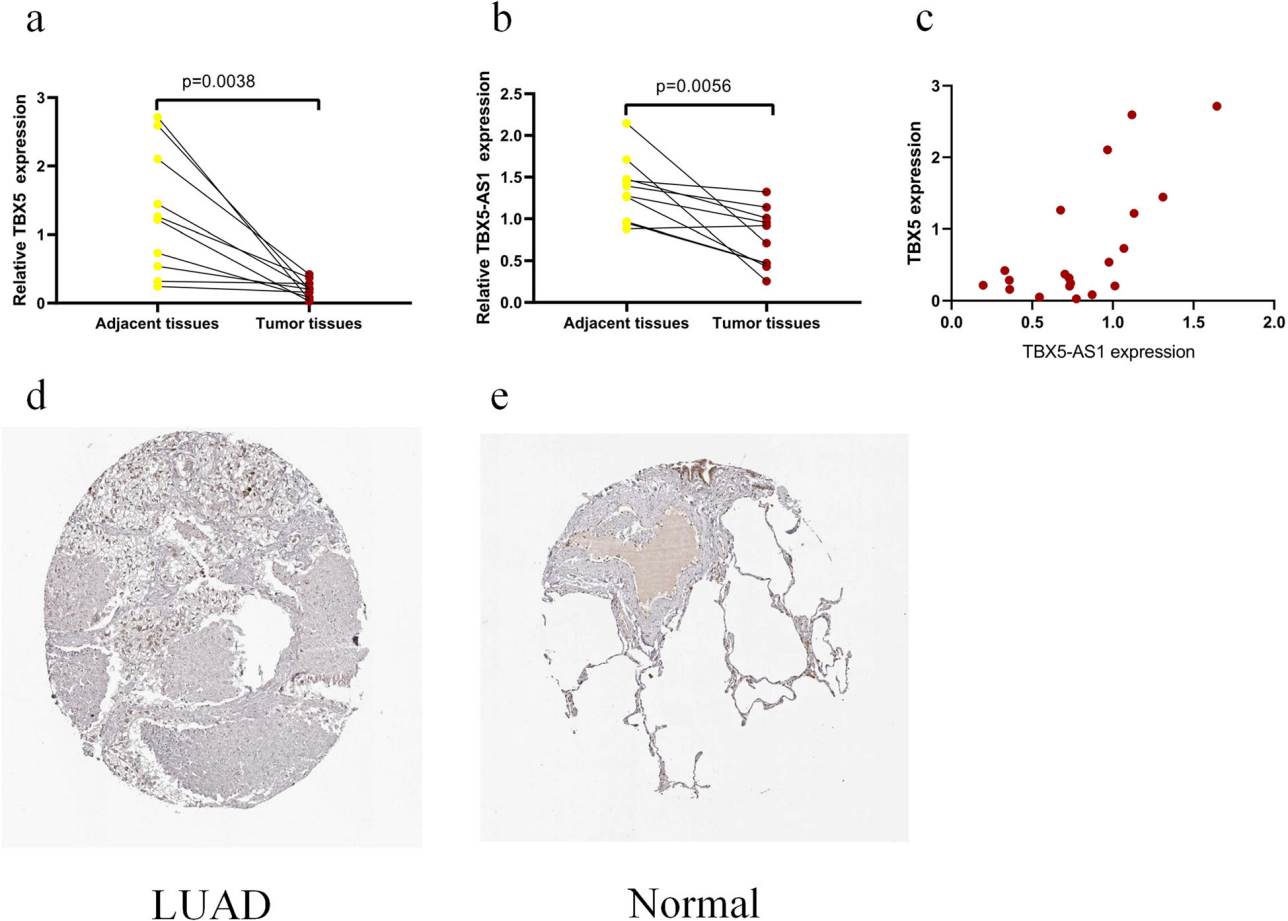
Quantiative reai-time polymerase chain reaction results for RNAs: **a** TBX5, **b** TBX5-AS1; **c** Correlation between TBX5 and TBX5-AS1; **d**-**e** *TBX5* expression in lung adenocarcinoma (LUAD) tissues and normal tissues from the Human Protein Atlas (HPA) database

qRT-PCR was used to measure the expression levels of TBX5-AS1 and TBX5 in 10 LUAD samples and paired adjacent samples. The outcomes indicated that the expression of TBX5-AS1 and TBX5 in LUAD tissues was significantly down-regulated when comparing it with adjacent tissues, which was consistent with its expression trend in the TCGA data set (*p* < 0.05; Fig. 4a-b). and a significantly positive correlation between TBX5-AS1 and TBX5 were seen in the samples (Spearman’s rank correlation coefficient *r* = 0.6893, *p* = 0.008) (Fig. 4c). Furthermore, immunohistochemistry data extracted from the HPA indicated that the protein expression of TBX5 was higher in non-tumour tissues compared with that in tumour tissues (Fig. 4d-e).

### Pan-Cancer verification

**Fig. 5:**
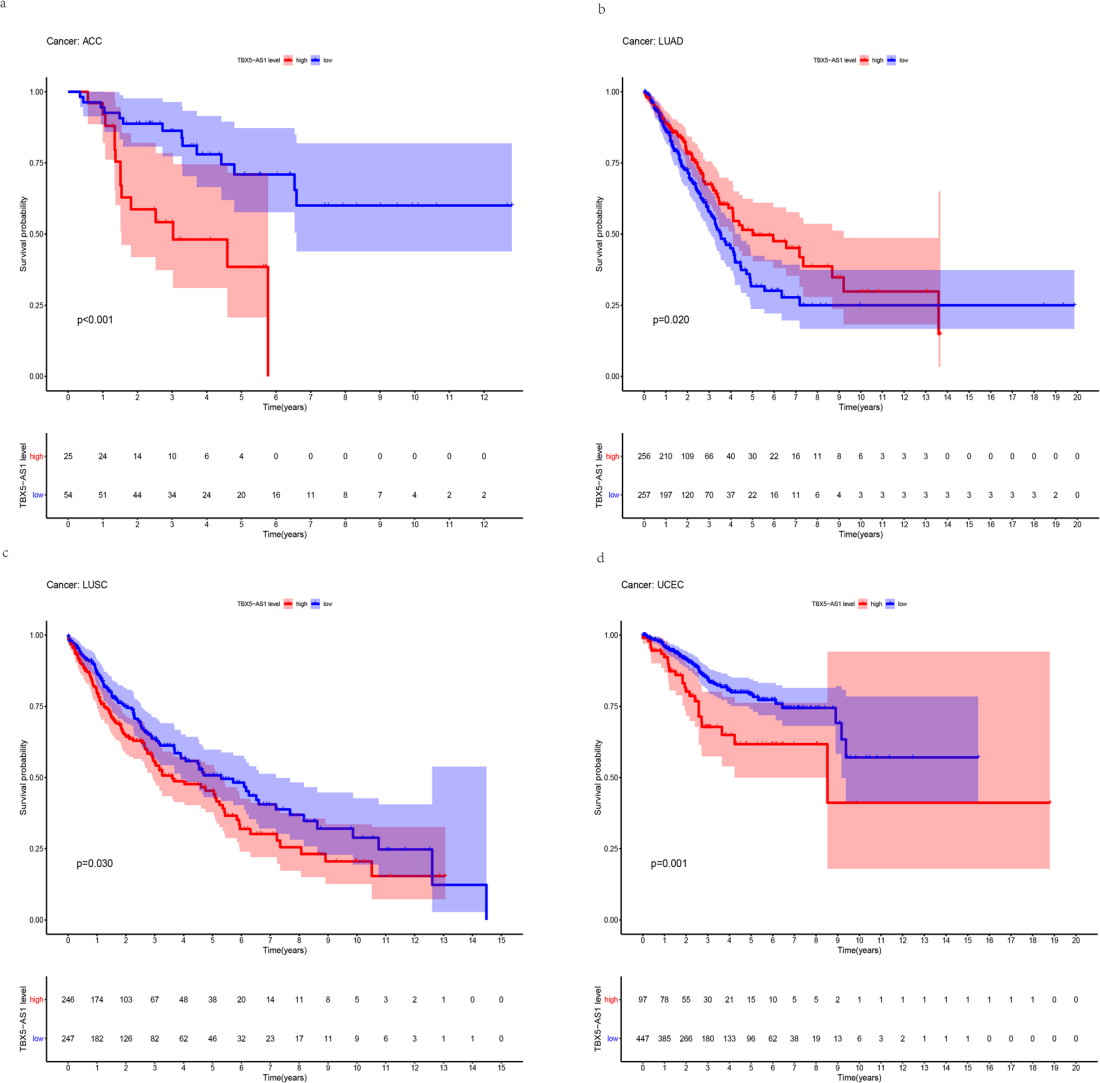
**a**–**d** Kaplan–Meier survival curves for TBX5-AS1 in pan-cancer (*p* < 0.05)

To determine the prognostic role of our selected eRNA in pan-cancer and the correlation with its target gene, we con-ducted survival and correlation analyses. The results showed that TBX5-AS1 was associated with survival in four tumours, namely, adrenocortical carcinoma (ACC), LUAD, uterine corpus endometrial carcinoma (UCEC) and lung squamous cell carcinoma (LUSC). The survival curves for TBX5-AS1 in these four tumours are shown in Fig. 5a–d. In addition, we found that TBX5-AS1 and its target gene were associated with 26 types of tumours (Additional file 1: Table S3).

## Discussion

Although it has been reported lncRNA TBX5-AS1 as antisense RNA of TBX5 could serve as potential diagnostic biomarkers for NSCLC and could inhibit the tumor progression of NSCLC through inactivating the PI3K/AKT pathway [27–29], the exact mechanism between TBX5-AS1 and TBX5 in LUAD has not been reported and there are no studies used eRNA as a biomarker for LUAD. In this study, we performed a comprehensive data analysis approach and found that TBX5-AS1 belong to a subclass of lncRNAs, termed “enhancer RNA”. Moreover, we also clarified the possible relationship between TBX5-AS1 and TBX5 and elaborated the involved pathways and possible molecular mechanism of TBX5-AS1 exert effects in LUAD.

eRNAs are noncoding RNAs that are transcribed from enhancers. Studies have shown that enhancers usually regulate the expression of target genes by forming a chromatin loop with the target promoter [8, 30]. For example, Rosenfeld et al. found that estrogen-regulated eRNAs can bind cohesin complex proteins, including RAD21 cohesin complex component (RAD21) and structural maintenance of chromosomes 3 (SMC3), and the authors proposed that eRNA/cohesin interactions serve to stabilize chromatin looping in breast cancer cells [31]. Additionally, mediator complex subunit 1 (MED1) in prostate cancer cells [21], heterogeneous nuclear ribonucleoprotein U (hnRNPU) in gastric cancer cells [19], CCCTC-binding factor (CTCF) in colon cancer cells [32], and mediator complex subunit 12 (MED12) in T-cell acute lymphoblastic leukaemia cells [33] have been shown to regulate chromatin loop stability through interactions with eRNAs, thereby modulating the expression of downstream target genes.

In the present study, we obtained RNA expression profile data and clinical information for LUAD from the TCGA database and integrated these data with eRNA-related information. Using Spearman’s correlation and Kaplan–Meier analyses, we identified 76 candidate eRNAs related to the prognosis of LUAD. We further identified TBX5-AS1 as the most critical eRNA candidate sequence in LUAD, and *TBX5* as its regulatory target. Clinical correlation analysis showed that *TBX5* was differentially expressed according to T stage, N stage, gender, and cancer status. KEGG pathway enrichment results suggested that *TBX5* may affect the survival outcome of LUAD patients through the PI3K/AKT pathway, Ras signaling pathway, cGMP−PKG signaling pathway, MAPK signaling pathway, etc. PI3K/AKT pathway is one of the most enriched biological process. Our pan-cancer validation results revealed that TBX5-AS1 was associated with survival in four types of tumours (ACC, LUAD, LUSC, and UCEC). Furthermore, TBX5-AS1 expression was correlated with that of its target gene, *TBX5*, in 26 tumour types. Finally, we validated the expression of TBX5-AS1 in surgical specimens by qRT-PCR and examined the expression levels of *TBX5* in LUAD using the HPA databases. Together, our results indicated that TBX5-AS1 could be used as an independent predictor of LUAD.

*TBX5,* a member of the T-box gene family, plays an important role in heart development [34–36]. Members of this family are involved in a variety of processes, including cell–cell signalling, proliferation, apoptosis, and migration [37]. Studies have shown that *TBX5* dysregulation plays an important role in breast cancer [37], gastric cancer [38], oesophageal carcinoma [39, 40], and colon cancer [41]. For example, the *TBX5* mRNA expression level is significantly downregulated in colorectal cancer cells; its methylation rate is significantly higher in cancer tissues than in normal tissues; and overexpressing *TBX5* can inhibit the growth of colorectal cancer cells, promote the apoptosis of cancer cells, and reduce the migration rate of cancer cells [41]. In this study, we found that *TBX5* was highly expressed in normal paracancerous tissues and cell lines, whereas its expression was downregulated in LUAD tissue and cells. These findings revealed that the level of *TBX5* is negatively correlated with lung cancer progression, which is consistent with the regulation of *TBX5* in colon cancer [41]. Besides, Ma et al. (2017) found that *TBX5* may induce early and late apoptosis in NSCLC cells by activating key molecules in the apoptotic pathway, including cleaved caspase-3, cleaved caspase-8, CDKN2, and PARP. Moreover, the overexpression of *TBX5* can inhibit cell proliferation, clone formation, and invasion, as well as induce apoptosis, in a NSCLC cell line [42]. To our knowledge, the specific interaction mechanism of TBX5-AS1 and TBX5 in LUAD has not been reported previously. Our results suggest that eRNA TBX5-AS1 might exert its antitumour effects through the following way: TBX5-AS1 may regulate chromatin loop stability by binding to specific protein factors, which in turn promotes the expression of its target gene *TBX5*. And then, *TBX5* suppress the invasion lung adenocarcinoma by affecting PI3K/AKT pathway, Ras signaling pathway, cGMP−PKG signaling pathway, MAPK signaling pathway, etc. In summary, the present findings suggested that TBX5-AS1 has potential as a diagnostic biomarker and therapeutic target for LUAD. In the future, in the early stage of disease, we could be detect the expression level of eRNA TBX5-AS1 in blood or other body fluids to determine whether the patients has LUAD. For patients who have been diagnosed with LUAD, we can predict the prognosis of lung adenocarcinoma patients based on TNM stage and the expression level of TBX5-AS1 in blood. This type of inspection would be particularly popular among patients with LUAD. Although lung biopsy histological examination is the gold standard for LUAD diagnosis and disease surveillance, histopathological examination is invasive, labour intensive and expensive which may impose a heavy financial burden on patients. Therefore, once our candidate biomarkers have been validated in blood and other body fluid samples, we can confirm or exclude lung adenocarcinoma through a simple and inexpensive blood test which would be significant.

Although we identified the key eRNA in LUAD and investigated its possible role in this disease, our study had several limitations. For instance, a larger clinical cohort is needed to further assess the diagnostic and prognostic potential of TBX5-AS1 in LUAD, while the potential function of TBX5-AS1 in LUAD also requires further investigation. Moreover, we demonstrated that TBX5-AS1 has roles in multiple cancer types, including ACC, LUAD, LUSC, and UCEC, and the emerging roles of TBX5-AS1 in these cancers also need to be characterized.

## Conclusion

In conclusion, we showed for the first time that TBX5-AS1 is a prognostic eRNA for LUAD, and through further prospective analysis, our study may help to evaluate prognosis of patients with LUAD, contribute to the early diagnosis of LUAD and provide treatment options for developing clinical interventions.

## Supplementary Information

**Additional file 1: Table S1.** List of 174 survival-related eRNAs in lung adenocarcinoma. **Table S2.** List of the 76 eRNAs with a significant correlation with their target gene in lung adenocarcinoma. **Table S3.** List of the 26 types of tumours associated with TBX5-AS1 and TBX5.

## Data Availability

The data supporting the findings of this study are available from the corresponding author on reasonable request. The datasets generated during the current study are available in the Cancer Genome Atlas (TCGA) http://portal.gdc.cancer.gov/), the The Human Protein Atlas (HPA) https://www.proteinatlas.org/).

## Abbreviations

ERNAs: Enhancer RNAs
LUAD: Lung adenocarcinoma
TCGA: The Cancer Genome Atlas
HPA: The human protein atlas
qRT-PCR: quantitative real time-polymerase chain reaction
TBX5: T-box transcription factor 5
ACC: Adrenocortical carcinoma
LUSC: Lung squamous cell carcinoma
UCEC: Uterine corpus endometrial carcinoma
NSCLC: Non-small cell lung cancer
T-ALL: T-cell acute lymphoblastic leukemia
HNSCC: Head and neck squamous cell carcinoma
KEGG: Kyoto Encyclopedia of Genes and Genomes
GO: Gene Ontology
MF: Molecular function
BP: Biological process
CC: Cellular component
SMC3: Structural maintenance of chromosomes 3
MED1: Mediator complex subunit 1
HnRNPU: Heterogeneous nuclear ribonucleoprotein U
