# Supplementary material for: TBX5-AS1, an enhancer RNA, is a potential novel prognostic biomarker for lung adenocarcinoma
Source: BMC Cancer. 2021 Jul 9;21:794. doi: 10.1186/s12885-021-08517-w (PMC8268367; doi:10.1186/s12885-021-08517-w)
Supplement: Supplementary file 1 — Additional file 1: Table S1. List of 174 survival-related eRNAs in lung adenocarcinoma. Table S2. List of the 76 eRNAs with a significant correlation with their target gene in lung adenocarcinoma. Table S3. List of the 26 types of tumours associated with TBX5-AS1 and TBX5. [file 12885_2021_8517_MOESM1_ESM.docx]

**Table S1.** List of 174 survival-related eRNAs in lung adenocarcinoma

| gene | | KM | FDR | gene | KM | FDR |
| --- | --- | --- | --- | --- | --- | --- |
| OGFRP1 | 4.36E-06 | | 0.000757982 | LINC02757 | 0.015363482 | 0.030377794 |
| LINC00518 | 3.28E-05 | | 0.002853053 | LINC01322 | 0.015486459 | 0.030276898 |
| PRKG1-AS1 | 3.56E-05 | | 0.002066022 | AL445430.1 | 0.015814072 | 0.030573872 |
| LINC01312 | 8.07E-05 | | 0.003511951 | AC012213.1 | 0.015831475 | 0.030271172 |
| LINC01833 | 0.000214583 | | 0.007467485 | LINC01798 | 0.015874666 | 0.030023825 |
| CMAHP | 0.000234485 | | 0.006800079 | SLC38A3 | 0.015913964 | 0.029774514 |
| LINC01031 | 0.000235838 | | 0.005862253 | LINC02577 | 0.015962633 | 0.029547853 |
| PRDM16-DT | 0.000299085 | | 0.006505093 | NDUFA6-DT | 0.016045747 | 0.029389053 |
| AL035587.1 | 0.000322413 | | 0.006233326 | AC064807.2 | 0.016054587 | 0.029098939 |
| LINC01128 | 0.000391053 | | 0.006804323 | AL161618.1 | 0.016500827 | 0.029599421 |
| AC022784.1 | 0.00042508 | | 0.006723996 | AC027117.1 | 0.016644391 | 0.029552285 |
| AL445430.2 | 0.000505265 | | 0.007326336 | AC108718.1 | 0.016704823 | 0.029359993 |
| AC090559.1 | 0.000513025 | | 0.006866641 | SOX2-OT | 0.016784065 | 0.029204273 |
| GAS1RR | 0.000580025 | | 0.007208879 | AL683813.2 | 0.016792067 | 0.028928908 |
| PAQR9-AS1 | 0.00058421 | | 0.006776841 | AL356421.2 | 0.016801751 | 0.02866181 |
| AC079760.2 | 0.000612412 | | 0.006659982 | NKAPP1 | 0.018499062 | 0.031250843 |
| AL158835.1 | 0.000618125 | | 0.006326687 | AC145285.2 | 0.018880712 | 0.031588883 |
| MIR34AHG | 0.000708131 | | 0.006845267 | AC093772.1 | 0.019217664 | 0.031846415 |
| LINC02613 | 0.000713919 | | 0.006537991 | AC022613.1 | 0.019330495 | 0.03173119 |
| CRNDE | 0.000844352 | | 0.007345863 | AC012618.3 | 0.01937068 | 0.031499984 |
| ZRANB2-AS2 | 0.000877338 | | 0.007269372 | AL359710.1 | 0.019461399 | 0.031354477 |
| AP003774.2 | 0.001106894 | | 0.008754523 | AC009226.1 | 0.019659406 | 0.031382904 |
| AC083805.1 | 0.00145923 | | 0.011039394 | TBX5-AS1 | 0.019877435 | 0.031442488 |
| AC121764.1 | 0.001470797 | | 0.010663281 | LRRC8C-DT | 0.020728017 | 0.032492567 |
| A2MP1 | 0.001651823 | | 0.011496689 | LINC02044 | 0.02095376 | 0.032553163 |
| LINC02657 | 0.001715687 | | 0.011481908 | JPX | 0.021461478 | 0.033046877 |
| AL451069.1 | 0.00179919 | | 0.011594781 | AP001347.1 | 0.022272736 | 0.033995229 |
| LHFPL3-AS2 | 0.002030719 | | 0.012619467 | LINC02365 | 0.022323758 | 0.033776817 |
| DRAIC | 0.002097119 | | 0.012582714 | AL137025.1 | 0.023364885 | 0.035047327 |
| FAM21EP | 0.002123556 | | 0.012316623 | CROCCP2 | 0.023799458 | 0.035394066 |
| LINC02390 | 0.002805842 | | 0.015748918 | AL096828.1 | 0.024096871 | 0.035532674 |
| LINC02812 | 0.002827859 | | 0.015376484 | AC093911.1 | 0.026562178 | 0.038838815 |
| LINC02723 | 0.002963114 | | 0.015623693 | HAR1A | 0.027133362 | 0.039343374 |
| AC010343.3 | 0.00311098 | | 0.015920899 | MIR646HG | 0.028019492 | 0.040292492 |
| TMEM210 | 0.003173815 | | 0.015778395 | C5orf66 | 0.028331786 | 0.040407629 |
| AC007255.1 | 0.003273213 | | 0.015820529 | CHRNA1 | 0.028745574 | 0.04066447 |
| NBDY | 0.003373322 | | 0.015863732 | AC105345.1 | 0.029106725 | 0.040843307 |
| AC079760.1 | 0.003506277 | | 0.016055059 | LINC02198 | 0.029538037 | 0.041116948 |
| AL035670.1 | 0.003517432 | | 0.015693157 | RAB30-DT | 0.029679294 | 0.040985691 |
| AC023796.1 | 0.003747343 | | 0.016300942 | ITFG2-AS1 | 0.02993094 | 0.041007744 |
| LINC01913 | 0.004112568 | | 0.017453339 | LINC01484 | 0.030032754 | 0.040825775 |
| LINC02611 | 0.004211541 | | 0.017447813 | ANKRD66 | 0.030070854 | 0.040560687 |
| GCC2-AS1 | 0.004488259 | | 0.018161791 | MIR583HG | 0.030283845 | 0.040533762 |
| GMDS-DT | 0.004518499 | | 0.017868609 | AC005013.1 | 0.030441897 | 0.040434276 |
| AL606491.1 | 0.004539423 | | 0.017552436 | HAGLR | 0.030741604 | 0.040523023 |
| LINC00996 | 0.004576694 | | 0.017311842 | LNCAROD | 0.031311008 | 0.040963274 |
| LRRC37A11P | 0.005116709 | | 0.018942711 | PCBP1-AS1 | 0.032138089 | 0.041731548 |
| AC124242.1 | 0.005189976 | | 0.018813663 | AP004608.1 | 0.033514414 | 0.043196356 |
| AC025871.2 | 0.005313697 | | 0.018869047 | LINC02265 | 0.033653459 | 0.043056631 |
| AC091849.2 | 0.005399954 | | 0.018791841 | LINC01238 | 0.034148331 | 0.043370873 |
| AC006357.1 | 0.006043434 | | 0.020618776 | AP5B1 | 0.034221175 | 0.043148437 |
| LINC00987 | 0.006057354 | | 0.02026884 | LINC02704 | 0.034271111 | 0.042900527 |
| DLGAP2 | 0.006068403 | | 0.019922682 | AL031846.1 | 0.034320865 | 0.042655932 |
| AC128709.2 | 0.006203926 | | 0.019990427 | PTGDS | 0.034679933 | 0.042796513 |
| FAM87A | 0.006241922 | | 0.019747172 | AL136369.2 | 0.034828362 | 0.042677007 |
| CT69 | 0.006286255 | | 0.019532294 | SEC24B-AS1 | 0.035330489 | 0.042989546 |
| AC012668.3 | 0.006716286 | | 0.020502348 | LINC01412 | 0.03584329 | 0.043310642 |
| LINC01150 | 0.008059146 | | 0.024177439 | AL450311.1 | 0.03599508 | 0.043194096 |
| AL589745.1 | 0.008086438 | | 0.02384814 | LINC02705 | 0.035999759 | 0.042903822 |
| LINC02422 | 0.008197772 | | 0.023773538 | IFNG-AS1 | 0.03600171 | 0.042614269 |
| AC012368.1 | 0.008325846 | | 0.023749134 | AP002992.1 | 0.03625731 | 0.042626837 |
| LINC02036 | 0.008884242 | | 0.024933195 | AC012485.1 | 0.03647091 | 0.04259019 |
| AC004923.1 | 0.008950581 | | 0.024720653 | LINC00460 | 0.038631341 | 0.044812355 |
| AL357500.1 | 0.009012177 | | 0.024501855 | LINC01615 | 0.03971243 | 0.045761343 |
| MIR4435-2HG | 0.009490702 | | 0.025405879 | AC105942.1 | 0.039747448 | 0.045500368 |
| AL160408.3 | 0.009605728 | | 0.025324192 | AP003721.2 | 0.040071979 | 0.045572055 |
| NBPF1 | 0.009852054 | | 0.025585932 | AC010931.1 | 0.040611521 | 0.045885744 |
| AP001972.3 | 0.009971921 | | 0.025516386 | CDK6-AS1 | 0.040638229 | 0.045619689 |
| AL691447.2 | 0.010660913 | | 0.026884041 | AL035252.3 | 0.041397724 | 0.046174384 |
| SLC2A1-AS1 | 0.010749764 | | 0.026720842 | AL138767.3 | 0.041640855 | 0.046149738 |
| LINC02739 | 0.010992644 | | 0.026939718 | AP003472.1 | 0.041767016 | 0.045996587 |
| AC005082.1 | 0.011373311 | | 0.027485502 | LINC-PINT | 0.042026882 | 0.045991682 |
| PINLYP | 0.011706549 | | 0.027903281 | AL390778.2 | 0.042550096 | 0.046273229 |
| AC106795.1 | 0.011966669 | | 0.028137843 | OSMR-AS1 | 0.04302328 | 0.04649721 |
| AC011379.2 | 0.012243007 | | 0.028403776 | AC111194.1 | 0.043123775 | 0.046318129 |
| LINC02572 | 0.01319193 | | 0.030202576 | LINC00926 | 0.043636478 | 0.046581271 |
| AL772337.2 | 0.013262465 | | 0.029969727 | SKINT1L | 0.044515459 | 0.047229817 |
| LINC01863 | 0.013580721 | | 0.030295455 | BAALC-AS1 | 0.044631234 | 0.047065665 |
| UCA1 | 0.013770143 | | 0.030329176 | AL035701.1 | 0.045332673 | 0.04751738 |
| AC008957.1 | 0.013969888 | | 0.030384505 | LINC02766 | 0.045981772 | 0.047909152 |
| AL139383.1 | 0.013980117 | | 0.030031362 | WT1-AS | 0.046418752 | 0.048076565 |
| LINC01891 | 0.01398964 | | 0.029685334 | AC021028.1 | 0.046518609 | 0.047894899 |
| KCP | 0.014141639 | | 0.029646327 | LINC01088 | 0.046821188 | 0.047922863 |
| AC090023.2 | 0.014145266 | | 0.029300907 | AP000424.1 | 0.046831284 | 0.047652885 |
| LINC02754 | 0.014296849 | | 0.029266491 | LINC00261 | 0.04683376 | 0.047378339 |
| FAM41C | 0.014726539 | | 0.029795557 | AL034397.2 | 0.048548355 | 0.048828981 |
| AC084375.1 | 0.01497338 | | 0.02994676 | AL031289.1 | 0.048607553 | 0.048607553 |

**Table S2.** List of the 76 eRNAs with a significant correlation with their target gene in lung adenocarcinoma

| eRNA | KM | FDR | Target | cor | corPval |
| --- | --- | --- | --- | --- | --- |
| CHRNA1 | 0.028745574 | 0.04066447 | CHRNA1 | 1 | 0 |
| TBX5-AS1 | 0.019877435 | 0.031442488 | TBX5 | 0.92524655 | 0.00E+00 |
| PRDM16-DT | 0.000299085 | 0.006505093 | PRDM16 | 0.902130238 | 1.98E-193 |
| LINC00261 | 0.04683376 | 0.047378339 | FOXA2 | 0.901811915 | 4.45E-193 |
| HAGLR | 0.030741604 | 0.040523023 | HOXD1 | 0.893699158 | 1.58E-184 |
| AC007255.1 | 0.003273213 | 0.015820529 | PRR15 | 0.880824592 | 2.74E-172 |
| WT1-AS | 0.046418752 | 0.048076565 | WT1 | 0.874029402 | 2.18E-166 |
| CRNDE | 0.000844352 | 0.007345863 | IRX5 | 0.800978307 | 0 |
| AC090559.1 | 0.000513025 | 0.006866641 | SPI1 | 0.782560711 | 0 |
| SOX2-OT | 0.016784065 | 0.029204273 | SOX2 | 0.754527106 | 0.00E+00 |
| LINC01615 | 3.97E-02 | 4.58E-02 | THBS2 | 0.736348025 | 5.88E-91 |
| PRKG1-AS1 | 3.56E-05 | 2.07E-03 | DKK1 | 0.724165496 | 1.30E-86 |
| LRRC8C-DT | 0.020728017 | 0.032492567 | LRRC8C | 0.719162447 | 0.00E+00 |
| AC091849.2 | 0.005399954 | 0.018791841 | LPCAT1 | 0.710118349 | 6.99E-82 |
| LINC00996 | 0.004576694 | 0.017311842 | GIMAP4 | 0.70748329 | 0.00E+00 |
| AL031846.1 | 0.034320865 | 0.042655932 | APOBEC3G | 0.695243314 | 3.58E-77 |
| BAALC-AS1 | 0.044631234 | 0.047065665 | FZD6 | 0.693071932 | 0 |
| LINC00996 | 0.004576694 | 0.017311842 | GIMAP7 | 0.691591836 | 0.00E+00 |
| GAS1RR | 0.000580025 | 0.007208879 | GAS1 | 0.687114245 | 1.02E-74 |
| MIR646HG | 0.028019492 | 0.040292492 | C20orf197 | 0.683039811 | 1.61E-73 |
| LINC01798 | 0.015874666 | 0.030023825 | MEIS1 | 0.673420566 | 9.17E-71 |
| LINC00996 | 0.004576694 | 0.017311842 | GIMAP8 | 0.667347937 | 0 |
| LINC00996 | 0.004576694 | 0.017311842 | GIMAP6 | 0.665025464 | 0 |
| PCBP1-AS1 | 0.032138089 | 0.041731548 | TIA1 | 0.658009971 | 0 |
| LINC00987 | 0.006057354 | 0.02026884 | A2M | 0.65730307 | 0.00E+00 |
| AC027117.1 | 0.016644391 | 0.029552285 | MTUS1 | 0.652571268 | 3.90E-65 |
| LINC01833 | 0.000214583 | 0.007467485 | SIX3 | 0.640706605 | 3.98E-62 |
| AL031846.1 | 0.034320865 | 0.042655932 | APOBEC3H | 0.635994793 | 5.73E-61 |
| AC124242.1 | 0.005189976 | 0.018813663 | ASAH1 | 0.6347887 | 0 |
| AC105942.1 | 0.039747448 | 0.045500368 | CNN3 | 0.632889806 | 0.00E+00 |
| AL035701.1 | 0.045332673 | 0.04751738 | ENPP4 | 0.607500009 | 2.26E-54 |
| AL031846.1 | 0.034320865 | 0.042655932 | APOBEC3D | 0.604121585 | 1.24E-53 |
| SLC2A1-AS1 | 0.010749764 | 0.026720842 | SLC2A1 | 0.576127898 | 0.00E+00 |
| AL138767.3 | 0.041640855 | 0.046149738 | PAPSS2 | 0.554979646 | 8.00E-44 |
| AC090023.2 | 0.014145266 | 0.029300907 | RPSAP52 | 0.548230974 | 1.34E-42 |
| A2MP1 | 0.001651823 | 0.011496689 | PZP | 0.540916348 | 2.63E-41 |
| AC091849.2 | 0.005399954 | 0.018791841 | SDHAP3 | 0.53987154 | 4.01E-41 |
| AC008957.1 | 0.013969888 | 0.030384505 | SLC1A3 | 0.538410769 | 7.19E-41 |
| JPX | 0.021461478 | 0.033046877 | XIST | 0.53161208 | 1.05E-39 |
| AP002992.1 | 0.03625731 | 0.042626837 | CHKA | 0.527988318 | 4.29E-39 |
| PCBP1-AS1 | 0.032138089 | 0.041731548 | ASPRV1 | 0.522194913 | 0.00E+00 |
| AL136369.2 | 0.034828362 | 0.042677007 | SFTA1P | 0.520547852 | 7.29E-38 |
| AL035701.1 | 0.045332673 | 0.04751738 | ENPP5 | 0.520078771 | 8.69E-38 |
| LINC00460 | 0.038631341 | 0.044812355 | EFNB2 | 0.51606976 | 3.88E-37 |
| LINC00987 | 0.006057354 | 0.02026884 | PZP | 0.508277837 | 6.70E-36 |
| NBPF1 | 0.009852054 | 0.025585932 | CROCCP2 | 0.503579999 | 0 |
| CROCCP2 | 0.023799458 | 0.035394066 | NBPF1 | 0.503579999 | 0.00E+00 |
| AL035670.1 | 0.003517432 | 0.015693157 | RCAN2 | 0.500283834 | 1.16E-34 |
| MIR646HG | 0.028019492 | 0.040292492 | CDH26 | 0.500214315 | 1.19E-34 |
| SKINT1L | 0.044515459 | 0.047229817 | SLC5A9 | 0.499353752 | 1.60E-34 |
| CDK6-AS1 | 0.040638229 | 0.045619689 | CDK6 | 0.497955762 | 2.62E-34 |
| AP000424.1 | 0.046831284 | 0.047652885 | RNF19A | 0.495319073 | 6.54E-34 |
| AC090023.2 | 0.014145266 | 0.029300907 | HMGA2 | 0.486134045 | 1.49E-32 |
| AP5B1 | 0.034221175 | 0.043148437 | SART1 | 0.478650638 | 0.00E+00 |
| AL136369.2 | 0.034828362 | 0.042677007 | CELF2 | 0.472026662 | 1.53E-30 |
| HAGLR | 0.030741604 | 0.040523023 | HOXD3 | 0.470320824 | 2.63E-30 |
| LINC02611 | 4.21E-03 | 1.74E-02 | MGAT4A | 0.469995908 | 0 |
| OGFRP1 | 4.36E-06 | 7.58E-04 | TCF20 | 0.462262218 | 0.00E+00 |
| AC012618.3 | 0.01937068 | 0.031499984 | ZNF563 | 0.462195467 | 3.38E-29 |
| AP004608.1 | 0.033514414 | 0.043196356 | B3GAT1 | 0.462164649 | 3.42E-29 |
| LINC02390 | 0.002805842 | 0.015748918 | CLECL1 | 0.458008221 | 1.23E-28 |
| LINC02705 | 0.035999759 | 0.042903822 | MS4A6A | 0.457999599 | 1.23E-28 |
| AP001972.3 | 0.009971921 | 0.025516386 | SLCO2B1 | 0.457573976 | 1.40E-28 |
| A2MP1 | 0.001651823 | 0.011496689 | A2M | 0.456976868 | 1.68E-28 |
| C5orf66 | 0.028331786 | 0.040407629 | PITX1 | 0.452131524 | 7.28E-28 |
| AL031846.1 | 0.034320865 | 0.042655932 | APOBEC3C | 0.443438408 | 9.52E-27 |
| LINC01088 | 0.046821188 | 0.047922863 | NAA11 | 0.440587137 | 2.18E-26 |
| IFNG-AS1 | 0.03600171 | 0.042614269 | IFNG | 0.437469584 | 5.33E-26 |
| AL031846.1 | 0.034320865 | 0.042655932 | APOBEC3F | 0.436580345 | 6.87E-26 |
| LINC01031 | 0.000235838 | 0.005862253 | B3GALT2 | 0.434351331 | 1.29E-25 |
| AP003472.1 | 0.041767016 | 0.045996587 | RNF19A | 0.426551594 | 1.14E-24 |
| AC025871.2 | 0.005313697 | 0.018869047 | FBXO16 | 0.426037153 | 1.31E-24 |
| LNCAROD | 0.031311008 | 0.040963274 | DKK1 | 0.419335459 | 8.14E-24 |
| AP001347.1 | 0.022272736 | 0.033995229 | RBM11 | 0.419266937 | 8.29E-24 |
| AL390778.2 | 0.042550096 | 0.046273229 | OLFM1 | 0.415573685 | 2.22E-23 |
| LINC02390 | 0.002805842 | 0.015748918 | CD69 | 0.402513675 | 6.62E-22 |

KM: P value obtained by Kaplan-Meier method; FDR: adjusted P value; corPval: correlation p value.

**Table S3.** List of the 26 types of tumours associated with TBX5-AS1 and TBX5

| **eRNA** | **Target** | **Cancer Type** | **cor** | **corPval** |
| --- | --- | --- | --- | --- |
| TBX5-AS1 | TBX5 | ACC | 0.5989106 | 5.53E-09 |
| TBX5-AS1 | TBX5 | BLCA | 0.8326764 | 4.94E-107 |
| TBX5-AS1 | TBX5 | BRCA | 0.8770332 | 0 |
| TBX5-AS1 | TBX5 | CESC | 0.4194789 | 1.80E-14 |
| TBX5-AS1 | TBX5 | CHOL | 0.5767645 | 0.000231373 |
| TBX5-AS1 | TBX5 | COAD | 0.2589931 | 1.17E-08 |
| TBX5-AS1 | TBX5 | DLBC | 0.7646364 | 2.52E-10 |
| TBX5-AS1 | TBX5 | ESCA | 0.9140766 | 0 |
| TBX5-AS1 | TBX5 | GBM | 0.9020273 | 1.87E-62 |
| TBX5-AS1 | TBX5 | HNSC | 0.6574639 | 1.87E-63 |
| TBX5-AS1 | TBX5 | KICH | 0.2249706 | 0.0715845 |
| TBX5-AS1 | TBX5 | KIRC | 0.5320754 | 1.95E-40 |
| TBX5-AS1 | TBX5 | KIRP | 0.1325354 | 0.024241102 |
| TBX5-AS1 | TBX5 | LAML | -0.091967 | 0.261395969 |
| TBX5-AS1 | TBX5 | LGG | 0.8853487 | 2.02E-177 |
| TBX5-AS1 | TBX5 | LIHC | 0.364035 | 3.66E-13 |
| TBX5-AS1 | TBX5 | LUAD | 0.9252466 | 0 |
| TBX5-AS1 | TBX5 | LUSC | 0.8997356 | 0 |
| TBX5-AS1 | TBX5 | MESO | 0.6877777 | 0 |
| TBX5-AS1 | TBX5 | OV | 0.7287158 | 5.77E-64 |
| TBX5-AS1 | TBX5 | PAAD | 0.6350192 | 1.75E-21 |
| TBX5-AS1 | TBX5 | PCPG | 0.6470103 | 4.38E-23 |
| TBX5-AS1 | TBX5 | PRAD | 0.9072072 | 5.03E-189 |
| TBX5-AS1 | TBX5 | READ | 0.1853615 | 0.016474638 |
| TBX5-AS1 | TBX5 | SARC | 0.8462399 | 2.50E-73 |
| TBX5-AS1 | TBX5 | SKCM | 0.8400256 | 1.24E-126 |
| TBX5-AS1 | TBX5 | STAD | 0.570027 | 1.07E-33 |
| TBX5-AS1 | TBX5 | TGCT | 0.7589281 | 1.72E-30 |
| TBX5-AS1 | TBX5 | THCA | 0.8033039 | 2.04E-116 |
| TBX5-AS1 | TBX5 | THYM | 0.8119393 | 4.01E-29 |
| TBX5-AS1 | TBX5 | UCEC | 0.3340499 | 9.44E-16 |
| TBX5-AS1 | TBX5 | UCS | 0.881578 | 3.03E-19 |
| TBX5-AS1 | TBX5 | UVM | 0.4291573 | 7.12E-05 |
